# As‐Needed Versus Regular Medication Use in the Treatment of Allergic Rhinitis in the Pollen Season: A MASK‐air Study

**DOI:** 10.1111/all.70508

**Published:** 2026-09-09

**Authors:** Bernardo Sousa‐Pinto, Rafael José Vieira, Antonio Bognanni, Ludger Klimek, Ana Margarida Pereira, Rita Amaral, Anna Bedbrook, Patrizia Bonadonna, Luisa Brussino, Mark S. Dykewicz, Mattia Giovannini, Bilun Gemicioglu, Juan Carlos Ivancevich, Violeta Kvedariene, Manuel Marques‐Cruz, Matteo Martini, André Moreira, Marek Niedoszytko, Michal Ordak, Nikolaos G. Papadopoulos, Oliver Pfaar, Nhân Pham‐Thi, Orathai Jirapongsananuruk, Frederico S. Regateiro, Sanna K. Toppila‐Salmi, Joaquin Sastre, Luís Taborda‐Barata, Ilgım Vardaloğlu Koyuncu, Arunas Valiulis, Maria Teresa Ventura, Kangsadarn Tanjararak, Yi‐Kui Xiang, Mohamed H. Shamji, Maria José Torres, Holger J. Schünemann, Danilo di Bona, João A. Fonseca, Torsten Zuberbier, Jean Bousquet

**Affiliations:** ^1^ MEDCIDS—Department of Community Medicine, Information and Health Decision Sciences University of Porto Porto Portugal; ^2^ CINTESISRISE—Centre for Health Technology and Services Research, Health Research Network University of Porto Porto Portugal; ^3^ Department of Health Research Methods, Evidence, and Impact and Department of Medicine McMaster University Hamilton Ontario Canada; ^4^ Clinical Epidemiology and Research Center (CERC) Humanitas University and Humanitas Research Hospital Milan Italy; ^5^ Department of Otolaryngology, Head and Neck Surgery Universitätsmedizin Mainz Mainz Germany; ^6^ Center for Rhinology and Allergology Wiesbaden Hesse Germany; ^7^ PaCeIT—Patient Centered Innovation and Technologies, Center for Health Technology and Services Research (CINTESIS) University of Porto Porto Portugal; ^8^ Allergy Unit Instituto and Hospital CUF Porto Portugal; ^9^ Department of Cardiovascular and Respiratory Sciences, Porto Health School Polytechnic Institute of Porto Porto Portugal; ^10^ Department of Women's and Children's Health, Paediatric Research Uppsala University Uppsala Sweden; ^11^ ARIA (Allergic Rhinitis and Its Impact on Asthma) Montpellier France; ^12^ MASK‐Air Montpellier France; ^13^ Allergy Unit Vicenza General Hospital ‐ ULSS 8 Vicenza Italy; ^14^ Department of Medical Sciences University of Torino Torino Italy; ^15^ Allergy and Clinical Immunology Unit Mauriziano Hospital Torino Italy; ^16^ Section of Allergy and Immunology Saint Louis University School of Medicine Saint Louis Missouri USA; ^17^ Allergy Unit Meyer Children's Hospital IRCCS Florence Italy; ^18^ Department of Health Sciences University of Florence Florence Italy; ^19^ Department of Pulmonary Diseases, Cerrahpaşa Istanbul University‐Cerrahpaşa Istanbul Turkey; ^20^ Department of Pulmonary Diseases Institute of Pulmonology and Tuberculosis, Istanbul University‐Cerrahpaşa Istanbul Turkey; ^21^ Santa Isabel Clinic, Head of Allergy and Immunology Buenos Aires Argentina; ^22^ Institute of Clinical Medicine, Clinic of Chest Diseases and Allergology Vilnius University Vilnius Lithuania; ^23^ Institute of Biomedical Sciences, Department of Pathology Vilnius University Vilnius Lithuania; ^24^ Allergy Unit, Department of Internal Medicine University Hospital A.O.U. Delle Marche Ancona Italy; ^25^ EPI Unit‐Institute of Public Health University of Porto, and Laboratory for Integrative and Translational Research in Population Health (ITR) Porto Portugal; ^26^ Serviço de Imunoalergologia Centro Hospitalar Universitário São João Porto Portugal; ^27^ Basic and Clinical Immunology Unit, Department of Pathology University of Porto Porto Portugal; ^28^ Department of Allergology Medical University of Gdansk Gdansk Poland; ^29^ Centre of Regenerative Medicine Medical University of Bialystok, Bialystok, Poland University of Warsaw Warsaw Poland; ^30^ Allergy Department Second Pediatric Clinic, University of Athens Athens Greece; ^31^ Section of Rhinology and Allergy, Department of Otorhinolaryngology, Head and Neck Surgery University Hospital Marburg, Philipps‐Universität Marburg Marburg Germany; ^32^ Ecole Polytechnique de Palaiseau Palaiseau France; ^33^ IRBA (Institut de Recherche Bio‐Médicale des Armées) Bretigny sur Orge France; ^34^ Université Paris Cité Paris France; ^35^ Center for Allergy and Clinical Immunology Paris France; ^36^ Division of Allergy and Immulogy, Department of Pediatrics Siriraj Hospital, Mahidol University Bangkok Thailand; ^37^ Allergy and Clinical Immunology Department Hospitais da Universidade de Coimbra, Unidade Local de Saúde de Coimbra Coimbra Portugal; ^38^ Center for Innovative Biomedicine and Biotechnology (CIBB) University of Coimbra Coimbra Portugal; ^39^ Institute of Immunology University of Coimbra Coimbra Portugal; ^40^ RISE‐Health and UBI Air—Clinical and Experimental Lung Centre University of Beira Interior Covilhã Portugal; ^41^ Department of Otorhinolaryngology University of Eastern Finland and the North Savo Wellbeing Services County, Joensuu and Kuopio Kuopio Finland; ^42^ Department of Allergology, Skin and Allergy Hospital, Inflammation Center Helsinki University Hospital and University of Helsinki Helsinki Finland; ^43^ Allergy Service, Fundacion Jimenez Diaz Universidad Autonoma de Madrid, CIBERES‐ISCIII Madrid Spain; ^44^ Department of Immunoallergology Unidade Local de Saúde Covilhã Portugal; ^45^ Department of Chest Diseases Bagcilar Training and Research Hospital Istanbul Turkey; ^46^ Human Ecology Interdisciplinary Research Group, Institute of Clinical Medicine and Institute of Health Sciences Medical Faculty of Vilnius University Vilnius Lithuania; ^47^ University of Bari Medical School Bari Italy; ^48^ Institute of Sciences of Food Production National Research Council (ISPA‐CNR) Bari Italy; ^49^ Department of Otolaryngology, Head & Neck Surgery, Ramathibodi Hospital Mahidol University Bangkok Thailand; ^50^ Charité – University Medical Center Berlin, Corporate Member of Freie Universität Berlin and Humboldt‐Universität zu Berlin, Institute of Allergology Berlin Germany; ^51^ Fraunhofer Institute for Translational Medicine and Pharmacology ITMP, Immunology and Allergology Berlin Germany; ^52^ Shanghai Skin Disease Hospital Tongji University School of Medicine Shanghai China; ^53^ National Heart and Lung Institute (NHLI) Imperial College London UK; ^54^ NIHR Imperial Biomedical Research Centre London UK; ^55^ Allergy Unit Regional University Hospital of Málaga, Málaga University, ARADyAL Málaga Spain; ^56^ Department of Medical and Surgical Science University of Foggia Foggia Italy

## Abstract

**Background:**

Patients with allergic rhinitis often use as‐needed medication instead of regular treatment. Using data from the MASK‐air mHealth app, we compared weeks with use of as‐needed versus regular medication on treatment satisfaction and symptom control.

**Methods:**

We assessed data from the MASK‐air app. We compared weeks in the pollen season in which patients reported the use of one same medication during all the days in the week (“regular treatment”) versus those in which patients reported the use of the same single medication (and no other treatments) in at most 4 days per week (“as‐needed treatment”). We built mixed‐effects linear regression models, evaluating the differences between regular and as‐needed treatments on visual analogue scales for symptom control and treatment satisfaction. We performed sensitivity analyses (i) considering a different definition of as‐needed treatment (use of medication ≤ 2 days per week), and (ii) evaluating treatment days preceded by non‐treatment days.

**Results:**

We assessed 2690 weeks (18,830 days, 1042 MASK‐air users). For intranasal corticosteroids (INCS) and fixed combinations of intranasal antihistamines (INAH) + INCS, there were no significant differences between as‐needed versus regular treatments for treatment satisfaction. For oral antihistamines (OAH) and INCS + OAH, results tended to favour as‐needed approaches. For symptom control, we observed either no significant differences between the two approaches or significant differences favouring as‐needed medication use. Similar results were found in the sensitivity analyses.

**Conclusion:**

Some patients use medication regularly and others use it on‐demand. This study shows that both approaches can satisfy patients and result in good disease control.

## Graphical abstract

- Using MASK‐air app data from 1042 users across 2690 weeks, this study compared the impact of regular versus as‐needed allergic rhinitis medication on symptom control and treatment satisfaction.
- Mixed‐effects linear regression models and sensitivity analyses revealed no significant differences between the two approaches, with results for oral antihistamines and combination therapies even tending to favour as‐needed use.
- Consequently, both regular and on‐demand treatment strategies effectively satisfy patients and yield good disease control.

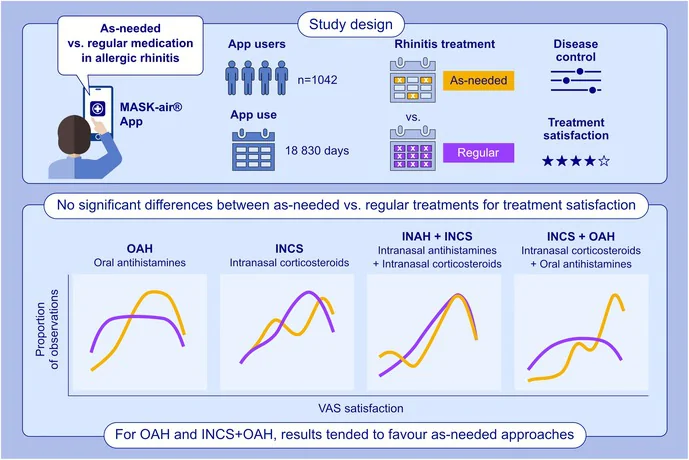

## 1 Introduction

The treatment of allergic rhinitis (AR) includes oral antihistamines (OAH), intranasal corticosteroids (INCS), and fixed combinations of intranasal antihistamines (INAH) + INCS [1]. So far, guideline recommendations for AR management have focused on the use of these medications on a regular (i.e., everyday) basis [2, 3], although as‐needed use has been recommended as a step‐down strategy once symptoms are controlled [4]. Most randomised controlled trials (RCTs) require an adherence ≥ 70% to the tested medications [5, 6]. By contrast, RCTs assessing the as‐needed use of medications are scarce and heterogeneous [7, 8, 9]. Nevertheless, observational studies suggest that most AR patients are not adherent to their treatments but rather use them according to their symptoms [10, 11, 12]. This lack of adherence is even observed among allergists with AR [13].

As‐needed treatment may be used partly because of AR variability: within the same period or season, the exposure can vary from day to day. Side effects with AR medication may also render some patients to use medication as‐needed. Considering how frequent the as‐needed use of rhinitis medication may be, it is important to determine whether this practice results in a worse level of disease control compared to that achieved when medications are used on a regular basis. In particular, it is relevant to evaluate whether patients report an adequate control of the disease even on days when medication is not used. In addition, it is important to assess if patients are satisfied with their treatments when they use them on an as‐needed basis. In fact, although partly associated to efficacy, treatment satisfaction does not only reflect how effective treatments are, but also other attributes. As a result, satisfaction is important in order to evaluate the acceptability of treatments [14].

In the absence of RCTs focused on as‐needed treatment, other sources of data can be explored. This is the case of mHealth, which has provided valuable evidence regarding patients' behaviour in relation to their symptoms and medication use [12] and patient satisfaction with different classes of AR medications [14]. Therefore, we performed a study using data directly provided by patients on the mHealth app MASK‐air in which we compared weeks in the pollen season when medication was used on an as‐needed basis versus on a regular basis on treatment satisfaction and symptom control. This study will inform the Allergic Rhinitis and its Impact on Asthma (ARIA)‐European Academy of Allergy and Clinical Immunology (EAACI) 2024–2025 guidelines [15, 16], in particular the question “Should continuous long‐term treatment vs. as‐needed treatment be used in patients with allergic rhinitis?” [17].

## 2 Methods

### 2.1 Study Design

This is an observational study using data directly provided by patients in the mHealth app MASK‐air. In our main analysis, we compared weeks in which patients reported medication use during all the days of the week (“regular treatment”) versus those in which patients reported medication use in at most 4 days per week (“as‐needed treatment”). In particular, these two treatment practices were compared in terms of treatment satisfaction and rhinitis symptoms. In addition, we compared different medication classes in terms of treatment satisfaction when used on a regular or an as‐needed basis. We explored the robustness of our results by means of sensitivity analyses (i) considering a different definition of as‐needed treatment (use of medication ≤ 2 days per week), and (ii) evaluating treatment days preceded by non‐treatment days.

### 2.2 Setting and Participants

MASK‐air (www.mask‐air.com) is an mHealth app freely available in 32 countries. It can be downloaded from the Google Play and Apple App Stores. MASK‐air is a Good Practice of the Directorate‐General for Health and Food Safety (European Commission) for digitally enabled patient‐centred care in rhinitis and asthma multimorbidity [18]. It is also a Best Practice of the Organisation for Economic Cooperation and Development (OECD) for Public Health on integrated care for chronic diseases [19].

We assessed MASK‐air data from European participants aged above 16 years (or ≥ 13 years, depending on the age of digital consent in each country [https://globaldatahub.taylorwessing.com/article/digital‐consent‐around‐the‐world]) with self‐reported AR (alone or with comorbid asthma). Data were reported between May 2015 and December 2025. However, data on treatment satisfaction were only available between January 2024 (when this question was introduced in MASK‐air) and December 2025. In our main analysis, we examined complete weeks (i.e., weeks with 7 days of MASK‐air data) during the pollen season. We considered weeks of “regular treatment” those in which patients reported using 7 days of OAH, INCS, fixed combinations of intranasal antihistamines (INAH) + INCS or INCS + OAH (defined as use of an INCS and an OAH on the same day). Importantly, we only included weeks in which patients had used medication from a single class (e.g., we included weeks in which patients used INCS for 7 days, but not weeks in which patients used INCS on some of the days and OAH [or INCS+OAH] on other days). We considered weeks of “as‐needed treatment” those in which patients reported using at most 4 days of OAH, INCS, INAH + INCS or INCS + OAH. As with regular treatment, we only included those weeks in which all medication used was from a single class. We analysed all days in those weeks on which the aforementioned eligibility criteria were met.

### 2.3 Ethics

MASK‐air is *Conformité Européene* (CE)‐registered and follows the European Union General Data Protection Regulation. We did not require an independent review board approval for this specific study because (i) the use of MASK‐air secondary data for research purposes has already been approved by an independent review board (Köln‐Bonn, Germany; reference number 17–069), (ii) all data were anonymised before the study using k‐anonymity, and (iii) users agreed to the analysis of their data for research purposes in the MASK‐air terms of use (translated into all languages and customised according to each country's legislation).

### 2.4 Data Sources and Variables

MASK‐air includes a daily monitoring questionnaire in which users report their rhinitis symptoms and medication use on that day. Users report their symptoms through four visual analogue scales (VASs), ranging from 0 to 100, with higher scores indicating a worse AR control (Table S1). These VASs have been assessed on their validity, reliability and responsiveness [20]. Rhinitis medication use is available from country‐specific lists with prescribed and over‐the‐counter medications. Importantly, when users report that they have used medication, they are asked on a VAS how satisfied they are with their AR treatment (“VAS satisfaction”; 0–100 scale, with higher scores indicating higher satisfaction) [14].

When responding to the MASK‐air daily monitoring questionnaire, it is not possible to skip any of the questions, and data are saved to the dataset only after the final answer. This precludes any missing data within each questionnaire.

### 2.5 Data Analysis

Categorical variables were described using absolute and relative frequencies, and continuous variables were described using medians and percentiles 25–75. Regression model results are presented with regression coefficients and 95% confidence intervals (CI). A significance level of 0.05 was adopted. All analyses were performed using software R (version 4.5.0) with packages lme4 and lmerTest.

In our main analysis, we compared days under regular treatment with those under as‐needed treatment in terms of VAS levels (median and maximum weekly levels) and, for each symptom VAS, proportion of days under full, good, medium and poor control (cut‐offs have been defined elsewhere [21]). In addition, for weeks under as‐needed treatment, we presented separate results for days when medication was used and those when medication was not used. To account for the existence of multiple observations per patient, we built mixed‐effects linear regression models setting the patient as a random effect (i.e., we clustered observations by patient), having the VAS as the dependent variable, and the treatment approach (as‐needed versus regular) as an independent variable. In addition to univariable models, we also built multivariable models, adjusting for patients' sex, age and baseline severity (ARIA score; range of 0–4; assesses the number of different ways in which allergy symptoms affect users). Importantly, we performed two additional sets of models: (i) one in which regular treatment days were compared with as‐needed days with medication use, and (ii) another in which regular treatment days were compared with as‐needed days with no medication use.

We performed additional analyses focused on comparing different medication classes in terms of VAS satisfaction. We built univariable and multivariable mixed‐effects regression models having VAS satisfaction as the dependent variable, the treatment class as the independent variable, and the patient as the random effect. In the multivariable models, we adjusted for patients' sex, age and baseline severity. We built such models separately for regular and for as‐needed medication use.

We performed sensitivity analyses focused on as‐needed medication use. In particular, we compared VAS levels in regular versus as‐needed treatment when the latter corresponded to the use of medication in at most 2 days per week. In addition, and in another sensitivity analysis, we identified days—from European or non‐European users and during or outside the pollen season—with medication use (OAH, INCS, INAH + INCS or INCS + OAH) preceded and followed by days without medication use. We compared the days when medication was used with those preceding them in terms of median VAS levels and proportion of days under each level of control. In addition, similarly to the main analysis, we built mixed‐effects linear regression models comparing different medication classes on VAS satisfaction.

## 3 Results

In the main analysis, we assessed 18,830 days (2690 weeks) reported by 1042 MASK‐air users. Less than half of the users were female (*N* = 490; 47.0%) and the average users' age was 39.1 years (SD = 14.2) (Table 1; Figure S1). The distribution of days and users per country is available in Table S2. We included 1496 weeks (55.6%) in which patients had used OAH, 502 (18.7%) of INCS use, 247 (9.2%) involving INAH + INCS, and 445 (16.5%) of INCS + OAH. Two‐thirds of the weeks (*N* = 1792; 66.6%) involved the use of medication on a regular basis. In fact, for all medication classes, we included a higher frequency of weeks of regular than of as‐needed treatment: this percentage ranged from 57.3% for OAH to 92.1% for INCS + OAH. Regarding weeks with use of as‐needed treatment, for all classes, most of the days involved the use of medication in at most 2 days per week (Table S3).

**TABLE 1:** Characteristics of the assessed participants and their reported days.

Variable	Assessed sample
*N* days	18,830
*N* users (average days per user)	1042 (18.1)
Females—*N* (%)	490 (47.0)
Age–mean (SD)	39.1 (14.2)
Asthma–*N* (%)	303 (29.1)
ARIA score–*N* (%)	
0	434 (42.8)
1	151 (14.9)
2	154 (15.2)
3	141 (13.9)
4	134 (13.2)
Medication classes used	
OAH	
*N* (%) weeks of regular treatment	857 (57.3)
*N* (%) weeks of as‐needed treatment	639 (42.7)
*N* days with medication use	10,472
INCS	
*N* (%) weeks of regular treatment	363 (72.3)
*N* (%) weeks of as‐needed treatment	139 (27.7)
*N* days with medication use	3514
INAH + INCS	
*N* (%) weeks of regular treatment	162 (65.6)
*N* (%) weeks of as‐needed treatment	85 (34.4)
*N* days with medication use	1729
INCS + OAH	
*N* (%) weeks of regular treatment	410 (92.1)
*N* (%) weeks of as‐needed treatment	35 (7.9)
*N* days with medication use	3115

Abbreviations: ARIA, Allergic Rhinitis and its Impact on Asthma; INAH, Intranasal antihistamines; INCS, Intranasal corticosteroids; OAH, Oral antihistamines; SD, standard‐deviation.

### 3.1 Rhinitis Treatment Satisfaction

The distribution of VAS satisfaction levels in as‐needed and regular treatment is displayed in Figure 1. For INCS and INAH + INCS, we observed a bimodal pattern in terms of treatment satisfaction when medication was used on an as‐needed basis (Figure 1). Nevertheless, there were no significant differences in terms of rhinitis treatment satisfaction in multivariable models comparing as‐needed versus regular medication use of these treatment classes (Table 2). On the other hand, differences favouring an as‐needed approach were observed for OAH (maximum VAS satisfaction) and INCS + OAH. In fact, for these medication classes, there was a relatively low proportion of observations in the regular approach that were associated with maximal satisfaction (Figure 1).

**FIGURE 1:**
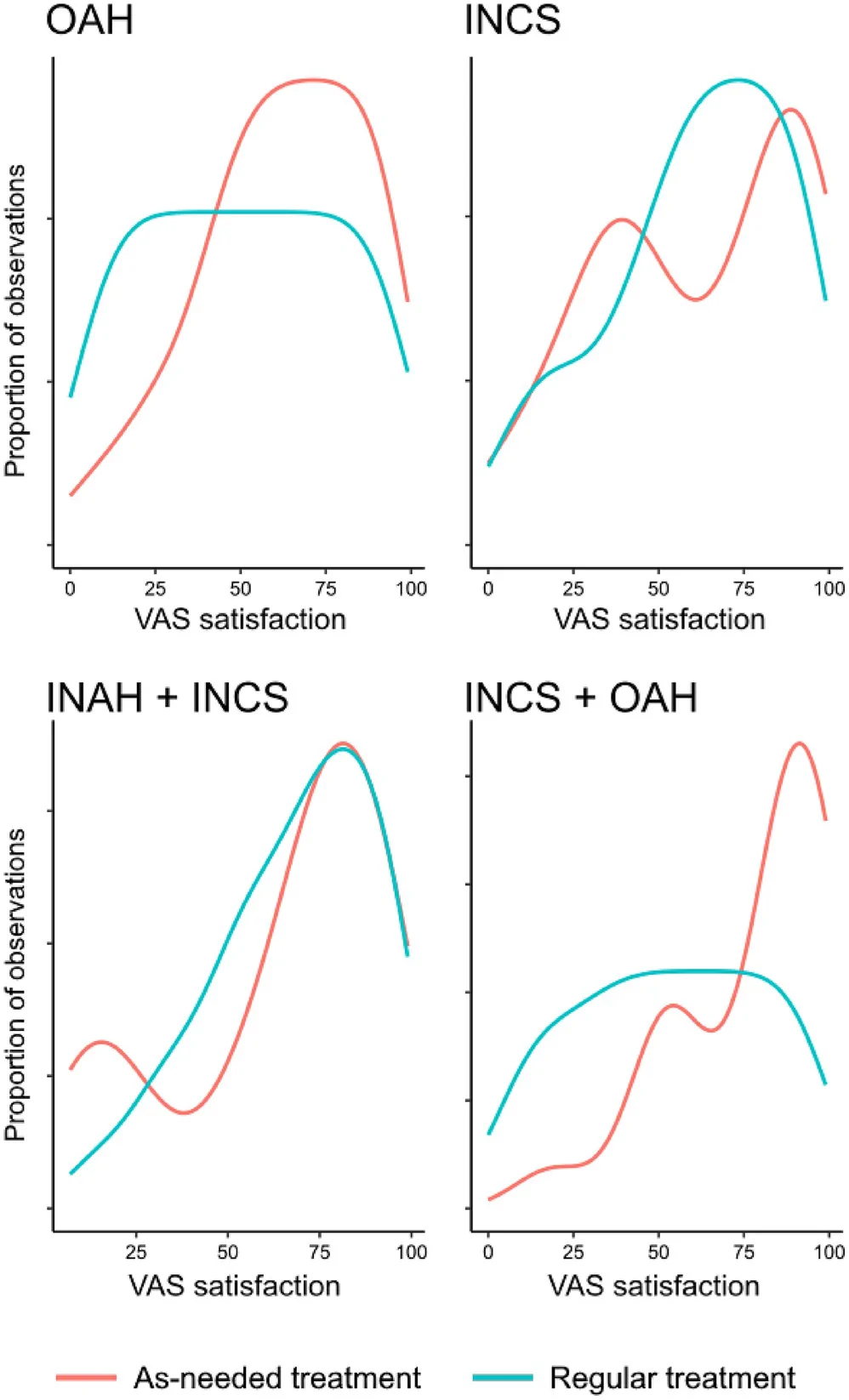
Distribution of the values of the visual analogue scale assessing patient satisfaction with rhinitis treatment (VAS satisfaction). INAH, Intranasal antihistamines; INCS, Intranasal corticosteroids; OAH, Oral antihistamines.

**TABLE 2:** Median and maximum visual analogue scale (VAS) levels in weeks of regular and as‐needed treatment.

A. Oral antihistamines				
Disease control	Regular treatment (*N* = 5999; 347 users)	As‐needed treatment (*N* = 4473 days; 341 users)	As‐needed vs regular medication use–coefficient (95% CI) [*p*]^a^	
			Unadjusted coefficients	Adjusted coefficients^b^
VAS global				
Median (IQR)	13 (4–29)	9 (2–22)	−6.02 (−7.21;4.83) [< 0.001]	−5.70 (−6.92;4.47) [< 0.001]
Maximum—median (IQR)	23 (10–49)	24 (11–49)	−2.25 (−5.13;0.62) [0.125]	−1.90 (−4.84;1.04) [0.206]
VAS nose				
Median (IQR)	13 (4–29)	10 (1–24)	−5.59 (−6.83;4.35) [< 0.001]	−5.16 (−6.44;3.88) [< 0.001]
Maximum—median (IQR)	24 (11–49)	26 (11–52)	−1.24 (−4.28;1.80) [0.424]	−0.92 (−4.02;2.17) [0.559]
VAS eye				
Median (IQR)	5 (0–17)	3 (0–12)	−4.04 (−5.03;3.04) [< 0.001]	−3.26 (−4.28;2.24) [< 0.001]
Maximum—median (IQR)	14 (4–32)	12 (3–32)	−1.04 (−3.67;1.60) [0.442]	−0.63 (−3.33;2.06) [0.645]
VAS satisfaction				
Median (IQR)	84 (62–93)	85 (66–94)	−0.74 (−3.55;2.06) [0.603]	0.73 (−2.23;3.68) [0.630]
Maximum—median (IQR)	76 (49–89)	83 (61–92)	6.98 (1.87;12.09) [0.008]	7.34 (2.04;12.64) [0.007]

B. Intranasal corticosteroids				
Disease control	Regular treatment (*N* = 2541 days; 121 users)	As‐needed treatment (*N* = 973 days; 85 users)	As‐needed vs regular medication use—coefficient (95% CI) [*p*]^a^	
			Unadjusted coefficients	Adjusted coefficients^b^
VAS global				
Median (IQR)	8 (3–21)	8 (2–16)	−1.88 (−3.20;0.55) [0.006]	−1.76 (−3.13;0.39) [0.012]
Maximum—median (IQR)	15 (7–30)	13 (5–28)	−2.76 (−6.33;0.81) [0.130]	−2.61 (−6.27;1.05) [0.163]
VAS nose				
Median (IQR)	11 (3–23)	9 (4–18)	−2.06 (−3.54;0.57) [0.007]	−1.88 (−3.42;0.34) [0.017]
Maximum—median (IQR)	19 (9–32)	18 (10–33)	−0.12 (−4.06;3.82) [0.953]	0.38 (−3.70;4.47) [0.854]
VAS eye				
Median (IQR)	3 (0–12)	3 (0–10)	−1.24 (−2.48;0.01) [0.048]	−1.23 (−2.50;0.03) [0.057]
Maximum—median (IQR)	8 (2–24)	9 (3–18)	−1.35 (−4.99;2.30) [0.469]	−1.25 (−5.02;2.52) [0.516]
VAS satisfaction				
Median (IQR)	88 (74–94)	93 (67–97)	−1.42 (−5.03;2.19) [0.440]	−1.51 (−5.35;2.32) [0.440]
Maximum—median (IQR)	84 (68–92)	90 (65–96)	2.42 (−3.86;8.69) [0.452]	2.25 (−4.66;9.16) [0.525]

C. Fixed combinations of intranasal antihistamines and corticosteroids				
Disease control	Regular treatment (*N* = 1134 days; 80 users)	As‐needed treatment (*N* = 595 days; 55 users)	As‐needed versus regular medication use—coefficient (95% CI) [*p*]^a^	
			Unadjusted coefficients	Adjusted coefficients^b^
VAS global				
Median (IQR)	10 (2–22)	7 (1–17)	−1.93 (−4.23;0.37) [0.100]	−1.96 (−4.26;0.33) [0.094]
Maximum—median (IQR)	19 (6–36)	15 (8–29)	−3.08 (−8.69;2.54) [0.284]	−3.16 (−8.77;2.44) [0.270]
VAS nose				
Median (IQR)	10 (1–22)	7 (0–16)	0.03 (−2.35;2.42) [0.978]	−0.02 (−2.40;2.37) [0.988]
Maximum—median (IQR)	19 (7–38)	17 (8–27)	−4.37 (−10.24;1.51) [0.146]	−4.55 (−10.42;1.32) [0.130]
VAS eye				
Median (IQR)	0 (0–4)	0 (0–7)	−0.93 (−2.54;0.68) [0.260]	−0.95 (−2.56;0.66) [0.249]
Maximum—median (IQR)	3 (0–11)	7 (1–15)	−0.51 (−3.94;2.92) [0.771]	−0.53 (−3.97;2.90) [0.760]
VAS satisfaction				
Median (IQR)	86 (74–94)	78 (66–87)	−6.16 (−22.22;9.90) [0.458]	−7.21 (−25.34;10.92) [0.442]
Maximum—median (IQR)	79 (66–92)	78 (66–90)	−1.17 (−20.59;18.24) [0.907]	−2.81 (−24.76;19.14) [0.804]

D. Intranasal corticosteroids and oral antihistamines				
Disease control	Regular treatment (*N* = 2870 days; 149 users)	As‐needed treatment (*N* = 245 days; 30 users)	As‐needed vs regular medication use–coefficient (95% CI) [*p*]^a^	
			Unadjusted coefficients	Adjusted coefficients^b^
VAS global				
Median (IQR)	11 (4–25)	9 (0–19)	−10.65 (−13.01;8.30) [< 0.001]	−10.59 (−12.99;8.18) [< 0.001]
Maximum—median (IQR)	18 (8–38)	23 (12–46)	−8.37 (−14.40;2.34) [0.007]	−8.41 (−14.63;2.20) [0.008]
VAS nose				
Median (IQR)	14 (5–26)	9 (2–24)	−9.02 (−11.65;6.39) [< 0.001]	−8.92 (−11.59;6.25) [< 0.001]
Maximum—median (IQR)	21 (10–41)	30 (17–53)	−3.97 (−10.70;2.78) [0.250]	−3.85 (−10.76;3.07) [0.276]
VAS eye				
Median (IQR)	5 (0–14)	2 (0–12)	−7.80 (−10.04;5.55) [< 0.001]	−7.70 (−9.96;5.44) [< 0.001]
Maximum—median (IQR)	11 (2–24)	12 (2–32)	−9.96 (−15.74;4.19) [< 0.001]	−9.97 (−15.88;4.07) [0.001]
VAS satisfaction				
Median (IQR)	87 (74–94)	92 (77–96)	9.84 (5.08;14.60) [< 0.001]	9.79 (4.75;14.84) [< 0.001]
Maximum—median (IQR)	83 (67–91)	83 (53–93)	11.58 (3.56;19.60) [0.005]	11.36 (2.81;19.91) [0.010]

Abbreviation: IQR, Interaquartile range.

^a^ Results of mixed‐effects linear regression models having the VAS as the dependent variable, the treatment approach as the independent variable, and the patient as a random effect. For VAS global, VAS nose and VAS eye, negative results are favourable to as‐needed treatment (i.e., indicate a decrease in symptoms with as‐needed treatment). For VAS satisfaction, positive results are favourable to as‐needed treatment (i.e., indicate an increase in satisfaction with as‐needed treatment).

^b^ Adjusted for age, sex and baseline severity.

When comparing different medication classes in terms of rhinitis treatment satisfaction, we observed that, when used on a regular basis, INAH + INCS were associated with higher treatment satisfaction than OAH (adjusted coefficient = 3.99; 95% CI = 2.83;5.15; *p* < 0.001). In addition, INCS were associated with higher satisfaction than INCS+OAH, when on regular use (adjusted coefficient = 0.86; 95% CI = 0.25;1.47; *p* = 0.005) (Table S4). On the other hand, no significant differences were observed when comparing different medication classes used on an as‐needed basis in terms of treatment satisfaction.

### 3.2 Rhinitis Symptoms

When comparing the levels of VAS for rhinitis symptoms, we observed either no significant differences between as‐needed and regular approaches or differences favouring as‐needed medication use (Table 2). These findings were obtained (i) both as a result of univariable and multivariable regression models, and (ii) for both median and maximum VAS levels. Overall, the largest differences between as‐needed and regular treatment were observed for OAH and INCS+OAH. In these classes, larger differences were observed for median than for maximum VASs.

Considering that an as‐needed approach encompasses days when medication was used and days when medication was not used, we built regression models separately comparing each of these two sets of days versus regular medication use (Tables 3 and 4). In multivariable models, except for INCS + OAH, we did not find any significant differences in VAS levels when comparing days of medication use on an as‐needed versus on a regular basis. For INCS + OAH, we observed results favouring the as‐needed approach. On the other hand, except for INAH + INCS (no significant differences), VAS levels registered on days when no medication was used were significantly lower than those observed when medication was used on a regular basis (Table 4).

**TABLE 3:** Visual analogue scale (VAS) levels and days under full, good, medium and poor control in weeks of regular and as‐needed treatment. In weeks of as‐needed treatments, results are separately provided for days when medication was used and for days when medication was not used.

A. Oral antihistamines				
Disease control^a^	Regular treatment (*N* = 5999; 347 users)	As‐needed treatment (*N* = 4473 days; 341 users)		
		All days	Days with medication use	Days with no medication use
VAS global–median (IQR)	13 (4–29)	9 (2–22)	14 (5–34)	7 (0–19)
Full control–*N* (%)	822 (13.7)	990 (22.1)	181 (13.3)	809 (26.0)
Good control–*N* (%)	3056 (50.9)	2249 (50.3)	653 (48.1)	1596 (51.2)
Medium control–*N* (%)	971 (16.2)	563 (12.6)	205 (15.1)	358 (11.5)
Poor control–*N* (%)	1150 (19.2)	671 (15.0)	319 (23.5)	352 (11.3)
VAS nose–median (IQR)	13 (4–29)	10 (1–24)	15 (5–33)	7 (0–20)
Full control–*N* (%)	856 (14.3)	1010 (22.6)	191 (14.1)	819 (26.3)
Good control–*N* (%)	2966 (49.4)	2153 (48.1)	627 (46.2)	1526 (49.0)
Medium control–*N* (%)	992 (16.5)	615 (13.7)	223 (16.4)	392 (12.6)
Poor control–*N* (%)	1185 (19.8)	695 (15.5)	317 (23.3)	378 (12.1)
VAS eye–median (IQR)	5 (0–17)	3 (0–12)	5 (0–17)	2 (0–10)
Full control–*N* (%)	1976 (32.9)	1885 (42.1)	491 (36.2)	1394 (44.8)
Good control–*N* (%)	2737 (45.6)	1891 (42.3)	587 (43.2)	1304 (41.9)
Medium control–*N* (%)	628 (10.5)	336 (7.5)	112 (8.2)	224 (7.2)
Poor control–*N* (%)	658 (11.0)	361 (8.1)	168 (12.4)	193 (6.2)

B. Intranasal corticosteroids				
Disease control^a^	Regular treatment (*N* = 2541 days; 121 users)	As‐needed treatment (*N* = 973 days; 85 users)		
		All days	Days with medication use	Days with no medication use
VAS global—median (IQR)	8 (3–21)	8 (2–16)	9 (2–18)	7 (1–15)
Full control—*N* (%)	486 (19.1)	212 (21.8)	64 (20.6)	148 (22.3)
Good control–*N* (%)	1383 (54.4)	573 (58.9)	178 (57.4)	395 (59.6)
Medium control—*N* (%)	469 (18.5)	92 (9.5)	37 (11.9)	55 (8.3)
Poor control—*N* (%)	203 (8.0)	96 (9.9)	31 (10.0)	65 (9.8)
VAS nose–median (IQR)	11 (3–23)	9 (4–18)	10 (5–21)	9 (3–17)
Full control–*N* (%)	512 (20.1)	169 (17.4)	54 (17.4)	115 (17.3)
Good control–*N* (%)	1298 (51.1)	586 (60.2)	178 (57.4)	408 (61.5)
Medium control–*N* (%)	490 (19.3)	94 (9.7)	37 (11.9)	57 (8.6)
Poor control–*N* (%)	241 (9.5)	124 (12.7)	41 (13.2)	83 (12.5)
VAS eye–median (IQR)	3 (0–12)	3 (0–10)	3 (0–10)	3 (0–10)
Full control–*N* (%)	1013 (39.9)	376 (38.6)	122 (39.4)	254 (38.3)
Good control–*N* (%)	1146 (45.1)	488 (50.2)	155 (50.0)	333 (50.2)
Medium control–*N* (%)	248 (9.8)	42 (4.3)	15 (4.8)	27 (4.1)
Poor control–*N* (%)	134 (5.3)	67 (6.9)	18 (5.8)	49 (7.4)

C. Fixed combinations of intranasal antihistamines and corticosteroids				
Disease control^a^	Regular treatment (*N* = 1134 days; 80 users)	As‐needed treatment (*N* = 595 days; 55 users)		
		All days	Days with medication use	Days with no medication use
VAS global–median (IQR)	10 (2–22)	7 (1–17)	10 (4–19)	6 (0–17)
Full control–*N* (%)	235 (20.7)	148 (24.9)	32 (16.2)	116 (29.2)
Good control–*N* (%)	600 (52.9)	333 (56.0)	122 (61.6)	211 (53.1)
Medium control–*N* (%)	146 (12.9)	72 (12.1)	21 (10.6)	51 (12.8)
Poor control–*N* (%)	153 (13.5)	42 (7.1)	23 (11.6)	19 (4.8)
VAS nose–median (IQR)	10 (1–22)	7 (0–16)	10 (4–18)	6 (0–15)
Full control–*N* (%)	247 (21.8)	159 (26.7)	37 (18.7)	122 (30.7)
Good control–*N* (%)	580 (51.1)	336 (56.5)	122 (61.6)	214 (53.9)
Medium control–*N* (%)	122 (10.8)	60 (10.1)	23 (11.6)	37 (9.3)
Poor control–*N* (%)	185 (16.3)	40 (6.7)	16 (8.1)	24 (6.0)
VAS eye–median (IQR)	0 (0–4)	0 (0–7)	2 (0–10)	0 (0–5)
Full control–*N* (%)	677 (59.7)	304 (51.1)	92 (46.5)	212 (53.4)
Good control–*N* (%)	377 (33.2)	238 (40.0)	80 (40.4)	158 (39.8)
Medium control–*N* (%)	26 (2.3)	37 (6.2)	17 (8.6)	20 (5.0)
Poor control–*N* (%)	54 (4.8)	16 (2.7)	9 (4.5)	7 (1.8)

D. Intranasal corticosteroids and oral antihistamines				
Disease control^a^	Regular treatment (*N* = 2870 days; 149 users)	As‐needed treatment (*N* = 245 days; 30 users)		
		All days	Days with medication use	Days with no medication use
VAS global–median (IQR)	11 (4–25)	9 (0–19)	10 (2–23)	8 (0–18)
Full control–*N* (%)	487 (17.0)	66 (26.9)	17 (23.0)	49 (28.7)
Good control–*N* (%)	1484 (51.7)	125 (51.0)	37 (50.0)	88 (51.5)
Medium control–*N* (%)	464 (16.2)	33 (13.5)	7 (9.5)	26 (15.2)
Poor control–*N* (%)	435 (15.2)	21 (8.6)	13 (17.6)	8 (4.7)
VAS nose–median (IQR)	14 (5–26)	9 (2–24)	12 (3–30)	9 (0–22)
Full control–*N* (%)	373 (13.0)	58 (23.7)	14 (19.4)	44 (25.7)
Good control–*N* (%)	1535 (53.5)	114 (46.5)	31 (43.1)	81 (47.4)
Medium control–*N* (%)	493 (17.2)	43 (17.6)	13 (18.1)	30 (17.5)
Poor control–*N* (%)	469 (16.3)	30 (12.2)	14 (19.4)	16 (9.4)
VAS eye–median (IQR)	5 (0–14)	2 (0–12)	2 (0–11)	2 (0–12)
Full control–*N* (%)	939 (32.7)	104 (42.4)	32 (43.2)	72 (42.1)
Good control–*N* (%)	1441 (50.2)	105 (42.9)	33 (44.6)	72 (42.1)
Medium control–*N* (%)	265 (9.2)	18 (7.3)	4 (5.4)	14 (8.2)
Poor control–*N* (%)	225 (7.8)	18 (7.3)	5 (6.8)	13 (7.6)

Abbreviation: IQR, Interquartile range.

^a^ VAS satisfaction levels not displayed in this table as patients only report VAS satisfaction on days when they use medication. Therefore, the median values of VAS satisfaction in regular and as‐needed (with medication use) days can be found in Table 2.

**TABLE 4:** Results of mixed‐effects linear regression models comparing as‐needed versus regular medication use on rhinitis symptoms^a^. For the as‐needed approach, results are separately provided for days when medication was used and for days when medication was not used.

	As‐needed with medication use versus regular—coefficient (95% CI) [*p*]	As‐needed with no medication use versus regular—coefficient (95% CI) [*p*]
**A. Oral antihistamines**		
Unadjusted results		
VAS global	−2.00 (−3.57;0.43) [0.013]	−7.65 (−8.87;6.43) [< 0.001]
VAS nose	−2.27 (−3.89;0.64) [0.006]	−7.07 (−8.34;5.80) [< 0.001]
VAS eye	−1.61 (−2.93;0.29) [0.017]	−4.94 (−5.97;3.91) [< 0.001]
Adjusted results^b^		
VAS global	−1.24 (−2.87;0.38) [0.133]	−7.53 (−8.79;6.28) [< 0.001]
VAS nose	−1.35 (−3.02;0.31) [0.111]	−6.88 (−8.18;5.58) [< 0.001]
VAS eye	−0.46 (−1.81;0.89) [0.503]	−4.39 (−5.44;3.34) [< 0.001]
**B. Intranasal corticosteroids**		
Unadjusted results		
VAS global	0.20 (−1.67;2.07) [0.833]	−2.62 (−4.08;1.17) [< 0.001]
VAS nose	0.07 (−2.01;2.15) [0.945]	−2.71 (−4.34;1.08) [0.001]
VAS eye	−1.07 (−2.85;0.72) [0.241]	−1.36 (−2.72;0) [0.050]
Adjusted results^b^		
VAS global	0.36 (−1.54;2.26) [0.712]	−2.52 (−4.02;1.02) [0.001]
VAS nose	0.27 (−1.85;2.40) [0.800]	−2.55 (−4.24;0.86) [0.003]
VAS eye	−1.03 (−2.84;0.79) [0.267]	−1.35 (−2.75;0.05) [0.059]
**C. Fixed combinations of intranasal antihistamines and corticosteroids**		
Unadjusted results		
VAS global	−0.96 (−4.18;2.26) [0.558]	−2.22 (−4.75;0.32) [0.087]
VAS nose	0.58 (−2.77;3.92) [0.736]	−0.28 (−2.94;2.38) [0.837]
VAS eye	−0.47 (−2.78;1.85) [0.693]	−0.92 (−2.73;0.89) [0.317]
Adjusted results^b^		
VAS global	−1.04 (−4.26;2.19) [0.529]	−2.27 (−4.81;0.26) [0.079]
VAS nose	0.47 (−2.88;3.81) [0.784]	−0.36 (−3.02;2.30) [0.790]
VAS eye	−0.51 (−2.83;1.81) [0.664]	−0.96 (−2.77;0.85) [0.300]
**D. Intranasal corticosteroids and oral antihistamines**		
Unadjusted results		
VAS global	−5.63 (−9.03;2.23) [0.001]	−12.67 (−15.27;10.08) [< 0.001]
VAS nose	−4.50 (−8.27;0.72) [0.020]	−10.50 (−13.40;7.60) [< 0.001]
VAS eye	−5.58 (−8.81;2.35) [< 0.001]	−8.31 (−10.83;5.78) [< 0.001]
Adjusted results^b^		
VAS global	−5.47 (−8.94;2.00) [0.002]	−12.60 (−15.25;9.95) [< 0.001]
VAS nose	−4.34 (−8.18;0.50) [0.027]	−10.36 (−13.31;7.41) [< 0.001]
VAS eye	−5.49 (−8.74;2.23) [0.001]	−8.19 (−10.74;5.65) [< 0.001]

Abbreviation: VAS, Visual analogue scale.

^a^ Results of mixed‐effects linear regression models having the VAS as the dependent variable, the treatment approach as the independent variable, and the patient as a random effect. Negative results are favourable to as‐needed treatment (i.e., indicate a decrease in symptoms with as‐needed treatment). VAS satisfaction levels not displayed in this table as patients only report VAS satisfaction in days when they use medication. Therefore, the median values of VAS satisfaction in regular and as‐needed (with medication use) days can be found in Table 2.

^b^ Adjusted for age, sex and baseline severity.

### 3.3 Sensitivity Analysis

Table S5 presents the results of the sensitivity analysis restricting weeks of as‐needed treatment to those in which patients used rhinitis medication at most 2 days per week. The results were consistent with those observed in the main analysis.

Tables 5 and S6 display the results of the sensitivity analysis restricted to as‐needed periods and comparing days in which medication was used versus those preceding them but without medication use. As in the main analysis, we observed lower VAS levels in days when medication was not used (Table 5). In terms of VAS satisfaction, in contradistinction with the main analysis, INCS were associated with higher satisfaction than OAH (adjusted coefficient = 3.28; 95% CI = 0.18;6.38; *p* = 0.038) and INCS + OAH (adjusted coefficient = 4.66; 95% CI = 0.15;9.17; *p* = 0.043) (Table S6).

**TABLE 5:** Results of the sensitivity analysis performed on days with medication use preceded and followed by days with no medication use (as‐needed approach): Visual analogue scale (VAS) levels and days under full, good, medium and poor control on days when medication was used and on the days before medication use (no medication use).

A. Oral antihistamines		
Disease control	Days with medication use (*N* = 4245 days; 1357 users)	Days before medication use (*N* = 4245 days; 1357 users)
VAS global—median (IQR)	16 (6–34)	9 (1–22)
Full control—*N* (%)	553 (13.0)	1004 (23.7)
Good control—*N* (%)	1964 (46.3)	2110 (49.7)
Medium control—*N* (%)	700 (16.5)	535 (12.6)
Poor control—*N* (%)	1028 (24.2)	596 (14.0)
VAS nose—median (IQR)	16 (5–37)	9 (1–24)
Full control—*N* (%)	629 (14.8)	1030 (24.3)
Good control—*N* (%)	1802 (42.4)	1970 (46.4)
Medium control—*N* (%)	710 (16.7)	540 (12.7)
Poor control—*N* (%)	1104 (26.0)	705 (16.6)
VAS eye—median (IQR)	4 (0–17)	2 (0–11)
Full control—*N* (%)	1574 (37.1)	1919 (45.2)
Good control—*N* (%)	1784 (42.0)	1716 (40.4)
Medium control—*N* (%)	384 (9.0)	311 (7.3)
Poor control—*N* (%)	503 (11.8)	299 (7.0)
VAS satisfaction—median (IQR)	81 (59–93)	—

B. Intranasal corticosteroids		
Disease control	Days with medication use (*N* = 1336 days; 528 users)	Days before medication use (*N* = 1336 days; 528 users)
VAS global—median (IQR)	10 (3–25)	7 (1–19)
Full control—*N* (%)	216 (16.2)	293 (21.9)
Good control—*N* (%)	714 (53.4)	739 (55.3)
Medium control—*N* (%)	188 (14.1)	132 (9.9)
Poor control—*N* (%)	218 (16.3)	172 (12.9)
VAS nose—median (IQR)	13 (6–30)	9 (3–21)
Full control—*N* (%)	164 (12.3)	266 (19.9)
Good control—*N* (%)	706 (52.8)	726 (54.3)
Medium control—*N* (%)	188 (14.1)	144 (10.8)
Poor control—*N* (%)	278 (20.8)	200 (15.0)
VAS eye—median (IQR)	3 (0–11)	2 (0–9)
Full control—*N* (%)	522 (39.1)	576 (43.1)
Good control—*N* (%)	620 (46.4)	600 (44.9)
Medium control—*N* (%)	89 (6.7)	82 (6.1)
Poor control—*N* (%)	105 (7.9)	78 (5.8)
VAS satisfaction—median (IQR)	90 (59–97)	—

C. Fixed combinations of intranasal antihistamines and corticosteroids		
Disease control	Days with medication use (*N* = 695 days; 242 users)	Days before medication use (*N* = 695 days; 242 users)
VAS global—median (IQR)	12 (5–25)	10 (2–21)
Full control—*N* (%)	93 (13.4)	144 (20.7)
Good control—*N* (%)	377 (54.2)	371 (53.4)
Medium control—*N* (%)	119 (17.1)	103 (14.8)
Poor control—*N* (%)	106 (15.3)	77 (11.1)
VAS nose—median (IQR)	14 (7–30)	12 (4–22)
Full control – *N* (%)	51 (7.3)	102 (14.7)
Good control—*N* (%)	391 (56.3)	400 (57.6)
Medium control–*N* (%)	118 (17.0)	99 (14.2)
Poor control–*N* (%)	135 (19.4)	94 (13.5)
VAS eye–median (IQR)	2 (0–12)	1 (0–10)
Full control–*N* (%)	311 (44.7)	331 (47.6)
Good control–*N* (%)	272 (39.1)	270 (38.8)
Medium control–*N* (%)	65 (9.4)	49 (7.1)
Poor control–*N* (%)	47 (6.8)	45 (6.5)
VAS satisfaction–median (IQR)	79 (54–90)	—

D. Intranasal corticosteroids and oral antihistamines		
Disease control	Days with medication use (*N* = 323 days; 187 users)	Days before medication use (*N* = 323 days; 187 users)
VAS global–median (IQR)	21 (7–46)	10 (0–30)
Full control–*N* (%)	43 (13.3)	87 (26.9)
Good control–*N* (%)	116 (35.9)	122 (37.8)
Medium control–*N* (%)	47 (14.6)	46 (14.2)
Poor control–*N* (%)	117 (36.2)	68 (21.1)
VAS nose–median (IQR)	24 (9–49)	11 (0–30)
Full control–*N* (%)	45 (13.9)	86 (26.6)
Good control–*N* (%)	94 (29.1)	121 (37.5)
Medium control–*N* (%)	62 (19.2)	46 (14.2)
Poor control–*N* (%)	122 (37.8)	70 (21.7)
VAS eye–median (IQR)	6 (0–27)	2 (0–17)
Full control–*N* (%)	127 (39.3)	150 (46.4)
Good control–*N* (%)	102 (31.6)	111 (34.4)
Medium control–*N* (%)	27 (8.4)	26 (8.0)
Poor control–*N* (%)	67 (20.7)	36 (11.1)
VAS satisfaction–median (IQR)	73 (54–89)	—

Abbreviation: IQR, Interquartile range.

## 4 Discussion

In this study, we used MASK‐air data to compare two different approaches for AR management: as‐needed versus regular use of medications of a single class. These approaches were compared on treatment satisfaction and symptoms. Both approaches displayed mostly comparable results in terms of treatment satisfaction. In addition, our results did not show the as‐needed approach to be associated with worse disease control when compared to regular treatment. We obtained consistent results in sensitivity analyses.

Overall, this study shows that when patients opt for an as‐needed approach with medication use of up to four times per week, they are able to achieve a control of their symptoms which is not worse than when medications are used on a regular basis. Importantly, the results of this study do not demonstrate that an as‐needed approach is more or as effective than one involving the regular use of medications. In fact, it is likely that there are differences in the severity of symptoms that may motivate patients to opt for one approach or the other (i.e., patients with more severe symptoms end up taking medication more often). However, this study suggests that most patients are capable of self‐managing their disease: in most cases where patients feel that as‐needed medication is appropriate, they do not achieve a worse level of disease control compared to a regular approach. This is particularly relevant, as patients commonly used medication on an on‐demand basis: in a previous MASK‐air study [11], among 11,713 European patients who reported at least 1 day of treatment with OAH, INCS or INAH+INCS, 1361 (11.6%) reported at least one full week of MASK‐air data in the pollen season (with medication adherence ranging from 31.7% to 38.5% depending on the treatment class), and only 484 (4.1%) reported at least one full month of MASK‐air data, with medication adherence ranging from 25.3% to 34.9%.

For some medication classes, OAH and INCS + OAH, treatment satisfaction was higher with as‐needed versus regular basis for reasons that are unclear. On the other hand, although differences were not significant, there was a trend for lower satisfaction when INAH + INCS were used on an as‐needed basis. This may be partly due to attributes such as their unpleasant taste and may explain why, for the as‐needed use of INAH + INCS (and contrary to what was observed for INCS), there was a bimodal distribution of observations: a larger “peak” of observations close to the maximum values of VAS satisfaction and a smaller one closer to the minimum values. Nevertheless, differences in VAS satisfaction may be inferior to the minimal important difference (MID). Although no formal study has calculated the minimal important difference for VAS satisfaction (particularly using anchor methods), a distribution‐based method of 0.5 × standard‐deviation would correspond to a MID of 12 points (out of 100) based on MASK‐air data (unpublished data). For the remaining VASs, most of our differences are also smaller than the VAS (11 points) [22].

Evidence comparing as‐needed versus regular use of AR medication is scarce. In a systematic review of RCTs to inform the ARIA 2024–2025 guidelines (in preparation), we identified few eligible studies, with heterogeneous designs and outcomes, and small sample sizes. In addition, these RCTs often compared as‐needed use of medication versus placebo (instead of versus their regular use). In this review, we did not find any significant results between the two approaches for INCS or INAH + INCS (although results non‐significantly tended to favour a regular approach) [8, 9, 10, 11, 12, 13, 14, 15, 16, 17, 18, 19, 20, 21, 22, 23, 24, 25]. For OAH, evidence was more heterogeneous, with some studies favouring a regular use of medication [7, 26] and others supporting an as‐needed approach [9]. Another aspect for which evidence is scarce concerns whether long‐term treatment has benefits in terms of nasal inflammation: the concepts of priming effect of the nasal mucosa [27] and of minimal persisting inflammation [28, 29] have been studied in nasal challenges performed in RCTs [30, 31]. However, in AR, there is no universally accepted treatment target analogous to the “treat‐to‐target” approach used in diseases like rheumatoid arthritis; that is, a specific strategy of titrating treatment until a predefined state of “minimal inflammation” is achieved has not been a major focus of AR.

This study has some limitations. First, for some analyses, only a small number of weeks met the eligibility criteria. This is particularly the case for the as‐needed use of INAH + INCS and INCS + OAH, for which the more limited amount of data has resulted in less precise estimates. This small number of weeks precluded the performance of paired analyses; that is, analyses comparing regular versus as‐needed treatment within the same patient. Second, there is a lack of generalisability regarding the proportion of weeks in which medication was used on a regular or an as‐needed basis. We cannot state that, in the general population, INCS + OAH are almost always used on a regular basis, and that OAH is the medication class most frequently used on an as‐needed basis. The results we observed mostly reflect the eligibility criteria we defined: (i) we considered only weeks occurring during the pollen season, and (ii) we did not consider weeks in which medication was not used on any of the days or weeks in which medication was used in 5 or 6 days. Importantly, we know from previous studies using MASK‐air data that patients using medication on an as‐needed basis frequently have weeks in which they do not use any treatment for rhinitis [32]. However, these weeks are systematically different from the remainder [32], and their inclusion would likely bias the results in favour of an as‐needed approach. In any case, the purpose of this study was not to determine the frequency in which each medication class is used on an as‐needed approach. Third, while we adjusted for participants' sex, age and ARIA score (“baseline severity”) in our multivariable models, there may still be relevant residual confounding. In particular, we were not able to account for the symptoms patients were experiencing in the days before the start of each week (a proxy of the severity of the symptoms during the studied periods), and which may differ between as‐needed and regular treatment weeks. For most weeks, such information was not available, and requiring it would result in an even more reduced sample size. Another variable for which information was not available—and for which, as a result, there may be some residual confounding—is that of patient behaviour. Although it is possible that patients more often taking treatment on a regular basis are more self‐aware and concerned about their disease, this does not accord with the poor adherence of allergists [13]. In any case, accounting for these behavioural differences would most likely bias the results more in favour of an as‐needed approach. Fourth, we measured treatment satisfaction using a single patient‐reported outcome measure corresponding to a VAS. While the VAS allows for the quantification of satisfaction in a simple and quick way [14], it does not inform about the reasons why patients may feel satisfied or unsatisfied about their treatments.

Finally, one aspect that merits discussion is that of the definition of regular and as‐needed treatment. As‐needed treatment was defined based on the use of medication on at most 4 days per week. The exclusion of weeks with 5 or 6 days of medication use is grounded in their similarity to weeks of regular treatment and in the difficulty to understand whether these correspond to “true weeks of as‐needed treatment” or rather as weeks of regular treatment in which the patient occasionally forgets to take or register his/her medication use. On the other hand, several patients treating themselves on an as‐needed basis use medication much less frequently than four times per week. As a result, we performed a sensitivity analysis in which as‐needed treatment was restricted to the use of medication for at most 2 days, and results were similar to the main analysis. Finally, for as‐needed weeks, and to ensure comparability with a regular strategy, we only considered weeks in which the same medication class was used on all treatment days. This may have led more favourably to as‐needed approaches (as less well‐controlled and more severe patients may tend to switch their medications more often [12]). However, our results are meant to concern AR management with a single medication regularly on an as‐needed basis, and are not to be generalisable for situations of treatment change or trial‐and‐error medication‐use approaches [12].

This manuscript also has some important strengths. This study is unique in its comparison between as‐needed versus regular medication use approaches. As discussed, patients frequently use medication on an as‐needed basis, but available evidence on that approach is scarce. Furthermore, this study not only evaluates AR symptoms but also treatment satisfaction that was found to be a new AR PROM. Importantly, the symptom VASs assessed in this study have been formally evaluated for their validity, reliability and responsiveness [20].

This study has direct relevance as it will inform the ARIA 2024–2025 guidelines [15, 16], which should reflect the practices followed by patients in order to be applicable. The ARIA 2024–2025 guidelines follow the GRADE approach [16]. As a result, recommendations are formulated based on the evidence available for a set of different criteria, in line with the Evidence‐to‐Decision framework. As a result, evidence from this study will need to be considered alongside the results of RCTs or other elements, such as the reduced costs and planetary health impacts (e.g., reduced carbon footprint or need of use of planet resources to produce medication) that are expected to stem from an as‐needed use of medication.

### 4.1 Generalisability

This study was performed using data from patients from 21 European countries. While across‐country differences may exist in terms of medication use and treatment satisfaction, there seems to be a mostly consistent pattern in terms of VAS satisfaction levels registered on an as‐needed or on a regular basis in individual countries (Table S7; we considered countries with at least 100 observations). MASK‐air users are perceived overall as more concerned about their health, digitally literate, and with better healthcare access than the general population with allergic rhinitis. However, these characteristics are expected to affect the users most often taking treatment as‐needed as well as those taking it on a regular basis (limiting the impact of these generalisability concerns in our findings).

## 5 Conclusion

This study suggests that when patients opt for an as‐needed approach, they can potentially achieve a symptom control that is comparable to that achieved when opting for regular treatment. In addition, treatment satisfaction does not seem to be inferior when AR medication is used as‐needed versus on a regular basis. Overall, it is important to consider that there may be relevant differences in the contexts leading patients to use medication regularly or as‐needed. Furthermore, the way patients use medication as‐needed may differ from the way this approach was defined in this study. That is, allergic rhinitis is variable between and within subjects and also depends on the level of allergen exposure. Some patients use medications regularly, and we showed that this approach satisfies the patient and can lead to adequate symptom control. Many patients use the treatment on‐demand, and this approach can also lead to patient satisfaction and good control levels. However, it must also be pointed out that both nonsedating OAH and current intranasal treatment options have an excellent safety profile also for long‐term use.

## Author Contributions

Bernardo Sousa‐Pinto and Rafael José Vieira participated in the study design, methodology, data analysis and manuscript writing. Antonio Bognanni, Matteo Martini, Manuel Marques‐Cruz, Danilo di Bona, Holger J. Schünemann and João A. Fonseca participated in the methodology and manuscript review. Jean Bousquet participated in the study design, methodology, and manuscript writing. All other authors participated in the inclusion of participants and manuscript review.

## Funding

This work has received funding from Breathing Together Onlus Association (Associazione Respiriamo Insieme Onlus), Italy; Clinical Research Support Program of the Japanese Society of Allergology, Japan; Espíritu Santo University, Samborondón, Ecuador; Finnish Anti‐Tuberculosis Association Foundation and Tampere Tuberculosis Foundation; State funding for Finnish university‐level health research (TYH2024309), Finland; Fundação ProAR, Brazil; Global Allergy and Asthma Excellence Network e.V., Germany; German Allergy Society AeDA (Ärzteverband Deutscher Allergologen), Germany; IPOKRaTES Lithuania Fund (International Postgraduate Organization for Knowledge transfer, Research and Teaching Excellent Students), Lithuania; Polish Society of Allergology (POLSKIE TOWARZYSTWO ALERGOLOGICZNE), Poland; University of Liège, Belgium; Neutec, Turkey.

## Conflicts of Interest

J. Bousquet reports personal fees from Cipla, Menarini, Mylan, Novartis, Purina, Sanofi‐Aventis, Teva, Noucor, other from KYomed‐Innov, other from Mask‐air‐SAS, outside the submitted work. L. Taborda‐Barata reports personal fees from LETI, personal fees from Sanofi, outside the submitted work. O. Pfaar reports grants and personal fees from ALK‐Abelló, grants and personal fees from Allergopharma, grants and personal fees from Stallergenes Greer, grants and personal fees from HAL Allergy Holding B.V./HAL Allergie GmbH, grants and personal fees from Bencard Allergie GmbH/Allergy Therapeutics, grants and personal fees from Laboratorios LETI/LETI Pharma, grants and personal fees from GlaxoSmithKline, personal fees from ROXALL Medizin, personal fees from Novartis, grants and personal fees from Sanofi‐Aventis and Sanofi‐Genzyme, personal fees from Med Update Europe GmbH, personal fees from streamedup! GmbH, personal fees from Pohl‐Boskamp, grants from Inmunotek S.L. personal fees from John Wiley and Sons, AS, personal fees from Paul‐Martini‐Stiftung (PMS), personal fees from Regeneron Pharmaceuticals Inc., personal fees from RG Aerztefortbildung, personal fees from Institut für Disease Management, personal fees from Springer GmbH, grants and personal fees from AstraZeneca, personal fees from IQVIA Commercial, personal fees from Ingress Health, personal fees from Wort&Bild Verlag, personal fees from Verlag ME, personal fees from Procter&Gamble, personal fees from ALTAMIRA, personal fees from Meinhardt Congress GmbH, personal fees from Deutsche Forschungsgemeinschaft, personal fees from Thieme‐Verlag, grants from Deutsche AllergieLiga e.V. personal fees from AeDA, personal fees from Alfried‐Krupp Krankenhaus, personal fees from Red Maple Trials Inc., personal fees from Königlich Dänisches Generalkonsulat, personal fees from Medizinische Hochschule Hannover, personal fees from ECM Expro and Conference Management, personal fees from Technical University Dresden, grants and personal fees from Lilly, personal fees from Japanese Society of Allergy, personal fees from Forum für Medizinische Fortbildung, personal fees from Dustri‐Verlag, personal fees from Pneumolive, grants and personal fees from ASIT Biotech, personal fees from LOFARMA, personal fees from Paul‐Ehrlich‐Institut, personal fees from Almirall, personal fees from BREAZY Health, personal fees from Biologix, personal fees from Blueprint, personal fees from CLIANTHA, from Cogitando GmbH/medcram, personal fees from Red Maple Trials, personal fees from Gemeinnütziger Verein zur Förderung der Allergiefortbildung (VFAF), outside the submitted work; and Vice President and member of EAACI Excom, member of ext. board of directors DGAKI; coordinator, main‐ or co‐author of different position papers and guidelines in rhinology, allergology and allergen‐immunotherapy; Editor‐in‐Chief (EIC) of Clinical Translational Allergy (CTA), Associate Editor (AE) of Allergy. T. Zuberbier reports personal fees from Amgen, personal fees from AstraZeneca, personal fees from AbbVie, personal fees from ALK‐Abelló, personal fees from Almirall, personal fees from Astellas, personal fees from Bayer Health Care, personal fees from Bencard, personal fees from Berlin Chemie, personal fees from FAES Farma, personal fees from HAL Allergie GmbH, personal fees from Henkel, personal fees from Kryolan, personal fees from Leti, personal fees from L'Oreal, personal fees from Meda, personal fees from Menarini, personal fees from Merck Sharp and Dohme, personal fees from Novartis, personal fees from Nuocor, personal fees from Pfizer, personal fees from Sanofi, personal fees from Stallergenes, personal fees from Takeda, personal fees from Teva, personal fees from UCB, personal fees from Uriach, personal fees from Abivax, personal fees from Blueprint, personal fees from Celldex, personal fees from Celltrion, outside the submitted work; and Committee member, “Allergic Rhinitis and its Impact on Asthma” (ARIA), Member of the Board, German Society for Allergy and Clinical Immunology (DGAKI), Head, European Centre for Allergy Research Foundation (ECARF), President, Global Allergy and Asthma Excellence Network (GA^2^LEN), and Member, Committee on Allergy Diagnosis and Molecular Allergology, World Allergy Organisation (WAO). L. Klimek reports personal fees from Allergopharma, personal fees from Viatris, personal fees from LETI Pharma, personal fees from Stallergenes, personal fees from ALK Abelló, personal fees from HAL Allergie, personal fees from Sanofi, personal fees from AstraZeneca, personal fees from GSK, personal fees from Inmunotek, personal fees from Regeneron Pharmaceuticals, personal fees from Novartis, outside the submitted work; and Membership: AeDA, DGHNO, Deutsche Gesellschaft für Allergologie und klinische Immunologie, DGHNO, HNO‐BV, GPA, EAACI. J. Sastre reports personal fees from GSK, personal fees from Gebro, grants from Sanofi, personal fees from Stallergenes outside the submitted work. M. Giovannini reports personal fees from Sanofi, Thermo Fisher Scientific, outside the submitted work. S. Toppila‐Salmi reports grants and other from Sanofi Pharma, grants and other from GSK, other from OrionPharma, other from ALK‐Abelló, other from AstraZeneca, other from Clario, outside the submitted work. N. Papadopoulos reports personal fees from Nestlé Nutrition Institute, personal fees from Abbott Nutrition, grants from Numil Hellas SA, grants from VIANEX, personal fees from GSK, personal fees from HAL Allergy Holding B.V, personal fees from Menarini International Operations Luxembourg SA, personal fees from Regeneron Pharmaceuticals Inc., personal fees from Berlin—Chemie AG, personal fees from DBV Technologies SA, grants from Vibrant America, personal fees from Hyproca Nutrition USA INC, personal fees from Danone Trading Medical B.V., personal fees from Med Maps srl, outside the submitted work. M. J. Torres reports personal fees from Leti Laboratories, personal fees from Aimmune Therapeutics, personal fees from Diater Laboratories, grants from European Commission, grants from ISCIII, grants from SEAIC, outside the submitted work. H. Schünemann reports and developed guidelines on Allergic Rhinitis in Asthma (ARIA) and his academic institution received research funding for it. The other authors have nothing to declare outside the submitted paper.

## Supporting information


**Table S1:** Visual analogue scales (VASs) available in MASK‐air.
**Table S2:** Frequency of MASK‐air users and daily monitoring questionnaire days per country.
**Table S3:** Frequency of weeks in which patients reported the use of 1, 2, 3 and 4 days of medication use (as‐needed treatment).
**Table S4:** Results of unadjusted and adjusted mixed‐effects linear regression models comparing rhinitis treatment classes on the levels of VAS satisfaction.
**Table S5:** Median and maximum visual analogue scale (VAS) levels in weeks of regular and as‐needed treatment: Sensitivity analysis with as‐needed treatment defined as use of medication of at most 2 days per week.
**Table S6:** Results of the sensitivity analysis performed in days with medication use preceded and followed by days with no medication use (as‐needed approach): Results of unadjusted and adjusted mixed‐effects linear regression models comparing rhinitis treatment classes on the levels of VAS satisfaction in an as‐needed context.
**Table S7:** Levels of the visual analogue scale (VAS) on treatment satisfaction in relation to oral antihistamines in individual countries. Countries were selected on the basis of having at least 100 observations of VAS satisfaction, something which was only observed for oral antihistamines.
**Figure S1:** Flow diagram illustrating participant selection.

## Data Availability

The data that support the findings of this study are available from the corresponding author upon reasonable request.
