# Supplementary material for: As‐Needed Versus Regular Medication Use in the Treatment of Allergic Rhinitis in the Pollen Season: A MASK‐air Study
Source: Allergy. 2026 Sep 9;81(11):4005–22. doi: 10.1111/all.70508 (PMC13753716; doi:10.1111/all.70508)
Supplement: Supplementary file 1 [file ALL-81-4005-s001.docx]

**Supplementary material**

**Supplementary Table 1. Visual analogue scales (VASs) available in MASK-air**

| **VAS** | **Question** |
| --- | --- |
| VAS Global (Allergy Symptoms) | Overall, how much are your allergic symptoms bothering you today? |
| VAS Nose | How much are your nose symptoms bothering you today? |
| VAS Eyes | How much are your eye symptoms bothering you today? |
| VAS Asthma | How much are your asthma symptoms bothering you today? |
| VAS Satisfaction | How satisfied are you with the treatment you have taken today for rhinitis? |

**Supplementary Table 2. Frequency of MASK-air^®^ users and daily monitoring questionnaire days per country**

| **Country** | ***N* days** | ***N* participants** |
| --- | --- | --- |
| Austria | 175 (0.9) | 12 (1.2) |
| Czech Republic | 791 (4.2) | 37 (3.6) |
| Finland | 238 (1.3) | 10 (1.0) |
| France | 1190 (6.3) | 67 (6.4) |
| Germany | 3024 (16.1) | 169 (16.2) |
| Great Britain | 140 (0.7) | 14 (1.3) |
| Greece | 987 (5.2) | 59 (5.7) |
| Hungary | 791 (4.2) | 38 (3.6) |
| Italy | 2051 (10.9) | 130 (12.5) |
| Lithuania | 2254 (12.0) | 101 (9.7) |
| Netherlands | 476 (2.5) | 29 (2.8) |
| Poland | 2247 (11.9) | 136 (13.1) |
| Portugal | 2366 (12.6) | 123 (11.8) |
| Slovenia | 476 (2.5) | 21 (2.0) |
| Spain | 1162 (6.2) | 64 (6.1) |
| Switzerland | 287 (1.5) | 17 (1.6) |
| Other countries | 175 (0.9) | 15 (1.4) |

**Supplementary Table 3. Frequency of weeks in which patients reported the use of one, two, three and four days of medication use (as-needed treatment)**

| ***N* weeks (%)** | **OAH** | **INCS** | **INAH+INCS** | **INCS+OAH** |
| --- | --- | --- | --- | --- |
| 1 medication day | 1855 (41.5) | 315 (32.4) | 196 (32.9) | 112 (45.7) |
| 2 medication days | 980 (21.9) | 287 (29.5) | 147 (24.7) | 35 (14.3) |
| 3 medication days | 861 (19.2) | 203 (20.9) | 112 (18.8) | 56 (22.9) |
| 4 medication days | 777 (17.4) | 168 (17.3) | 140 (23.5) | 42 (17.1) |

INAH: Intranasal antihistamines; INCS: Intranasal corticosteroids; OAH: Oral antihistamines

**Supplementary Table 4. Results of unadjusted and adjusted mixed-effects linear regression models comparing rhinitis treatment classes on the levels of VAS satisfaction**

|  | **Regular treatment – coefficient (95%CI) [*p*-value]** | **As-needed treatment – coefficient (95%CI) [*p*-value]** |
| --- | --- | --- |
| **A. Unadjusted models** | | |
| OAH vs INCS | -0.62 (-1.63;0.39) [0.227] | 1.60 (-0.12;3.31) [0.069] |
| OAH vs INAH+INCS | -3.85 (-4.98;-2.72) [<0.001] | 1.55 (-0.28;3.38) [0.099] |
| INCS vs INAH+INCS | 0.08 (-0.84;1.00) [0.867] | 0.61 (-2.24;3.47) [0.675] |
| OAH vs INCS+OAH | -0.42 (-0.83;-0.01) [0.052] | 1.41 (-0.41;3.23) [0.132] |
| INCS vs INCS+OAH | 0.84 (0.27;1.41) [0.004] | 0 (-3.10;3.10) [0.997] |
| INAH+INCS vs INCS+OAH | 0.30 (-1.35;1.95) [0.722] | -3.16 (-7.33;1.01) [0.142] |
| **B. Adjusted models ^a^** | | |
| OAH vs INCS | -0.70 (-1.72;0.31) [0.196] | 1.60 (-0.17;3.37) [0.077] |
| OAH vs INAH+INCS | -3.99 (-5.15;-2.83) [<0.001] | 1.41 (-0.47;3.29) [0.143] |
| INCS vs INAH+INCS | 0.09 (-0.87;1.04) [0.860] | 0.41 (-2.54;3.35) [0.786] |
| OAH vs INCS+OAH | -0.42 (-0.85;0.01) [0.060] | 1.38 (-0.48;3.24) [0.149] |
| INCS vs INCS+OAH | 0.86 (0.25;1.47) [0.005] | -0.27 (-3.33;2.79) [0.862] |
| INAH+INCS vs INCS+OAH | 0.58 (-1.13;2.29) [0.506] | -2.96 (-7.23;1.31) [0.179] |

INAH=Intranasal antihistamines; INCS=Intranasal corticosteroids; OAH=Oral antihistamines; VAS satisfaction=Visual analogue scale evaluating patients’ satisfaction with their rhinitis treatment; ^a^ Adjusted for age, sex and baseline severity.

**Supplementary Table 5. Median and maximum visual analogue scale (VAS) levels in weeks of regular and as-needed treatment: Sensitivity analysis with as-needed treatment defined as use of medication of at most two days per week**

| **A. Oral antihistamines** |  |  |  |  |
| --- | --- | --- | --- | --- |
| **Disease control** | **Regular treatment**  **(*N*=5999; 347 users)** | **As-needed treatment**  **(*N*=2835 days; 255 users)** | **As-needed *vs* regular medication use – coefficient (95%CI) [*p*-value] ^a^** | |
|  |  |  | **Unadjusted coefficients** | **Adjusted coefficients ^b^** |
| VAS global – median (IQR) | 13 (4-29) | 8 (1-21) | -7.13 (-8.54;-5.71) [<0.001] | -7.36 (-8.81;-5.91) [<0.001] |
| VAS nose – median (IQR) | 13 (4-29) | 8 (1-22) | -6.17 (-7.64;-4.70) [<0.001] | -6.37 (-7.87;-4.87) [<0.001] |
| VAS eye – median (IQR) | 5 (0-17) | 2 (0-12) | -4.01 (-5.18;-2.83) [<0.001] | -4.06 (-5.26;-2.86) [<0.001] |
| VAS satisfaction – median (IQR) | 84 (62-93) | 81 (65-93) | -1.91 (-6.17;2.36) [0.381] | -1.85 (-6.39;2.69) [0.425] |
| **B. Intranasal corticosteroids** | |  |  |  |
| **Disease control** | **Regular treatment**  **(*N*=2541 days; 121 users)** | **As-needed treatment**  **(*N*=602 days; 60 users)** | **As-needed *vs* regular medication use – coefficient (95%CI) [*p*-value] ^a^** | |
|  |  |  | **Unadjusted coefficients** | **Adjusted coefficients ^b^** |
| VAS global – median (IQR) | 8 (3-21) | 8 (2-18) | -2.57 (-4.14;-1.00) [0.001] | -2.46 (-4.10;-0.82) [0.003] |
| VAS nose – median (IQR) | 11 (3-23) | 10 (5-21) | -2.75 (-5.38;-0.12) [0.002] | -2.61 (-4.48;-0.78) [0.005] |
| VAS eye – median (IQR) | 3 (0-12) | 3 (0-11) | -0.86 (-2.36;0.64) [0.260] | -0.80 (-2.36;0.76) [0.315] |
| VAS satisfaction – median (IQR) | 88 (74-94) | 96 (3-65) | -1.02 (-6.02;3.98) [0.690] | -1.01 (-6.46;4.43) [0.715] |
| **C. Fixed combinations of intranasal antihistamines and corticosteroids** | | |  |  |
| **Disease control** | **Regular treatment**  **(*N*=1134 days; 80 users)** | **As-needed treatment**  **(*N*=343 days; 37 users)** | **As-needed *vs* regular medication use – coefficient (95%CI) [*p*-value] ^a^** | |
|  |  |  | **Unadjusted coefficients** | **Adjusted coefficients ^b^** |
| VAS global – median (IQR) | 10 (2-22) | 5 (0-14) | -2.50 (-5.42;0.42) [0.094] | -2.51 (-5.43;0.41) [0.092] |
| VAS nose – median (IQR) | 10 (1-22) | 5 (0-13) | -0.66 (-3.76;2.43) [0.675] | -0.71 (-3.81;2.38) [0.653] |
| VAS eye – median (IQR) | 0 (0-4) | 0 (0-4) | -0.62 (-2.63;1.39) [0.546] | -0.64 (-2.66;1.37) [0.534] |
| VAS satisfaction – median (IQR) | 86 (74-94) | 89 (81-93) | 5.09 (-9.36;19.54) [0.495] | 0.54 (-16.81;17.89) [0.952] |
| **D. Intranasal corticosteroids and oral antihistamines** | | |  |  |
| **Disease control** | **Regular treatment**  **(*N*=2870 days; 149 users)** | **As-needed treatment**  **(*N*=147 days; 20 users)** | **As-needed *vs* regular medication use – coefficient (95%CI) [*p*-value] ^a^** | |
|  |  |  | **Unadjusted coefficients** | **Adjusted coefficients ^b^** |
| VAS global – median (IQR) | 11 (4-25) | 10 (1-21) | -14.6 (-17.6;-11.5) [<0.001] | -14.5 (-17.7;-11.4) [<0.001] |
| VAS nose – median (IQR) | 14 (5-26) | 11 (1-29) | -12.2 (-15.6;-8.75) [<0.001] | -12.1 (-15.6;-8.66) [<0.001] |
| VAS eye – median (IQR) | 5 (0-14) | 5 (0-16) | -7.76 (-10.7;-4.80) [<0.001] | -7.76 (-10.8;-4.77) [<0.001] |
| VAS satisfaction – median (IQR) | 87 (74-94) | 85 (53-93) | 0.07 (-6.82;6.97) [0.983] | 0.14 (-7.16;7.45) [0.969] |

IQR=Interaquartile range; ^a^ Results of mixed-effects linear regression models having the VAS as the dependent variable, the treatment approach as the independent variable, and the patient as a random effect. For VAS global, VAS nose and VAS eye, negative results are favourable to as-needed treatment (i.e., indicate a decrease in symptoms with as-needed treatment). For VAS satisfaction, positive results are favourable to as-needed treatment (i.e., indicate an increase in satisfaction with as-needed treatment); ^b^ Adjusted for age, sex and baseline severity.

**Supplementary Table 6. Results of the sensitivity analysis performed in days with medication use preceded and followed by days with no medication use (as-needed approach): Results of unadjusted and adjusted mixed-effects linear regression models comparing rhinitis treatment classes on the levels of VAS satisfaction in an as-needed context.**

|  | **As-needed treatment – coefficient (95%CI) [*p*-value]** |
| --- | --- |
| **A. Unadjusted models** |  |
| OAH vs INCS | -2.99 (-5.99;0.02) [0.052] |
| OAH vs INAH+INCS | 0.85 (-3.50;5.20) [0.702] |
| INCS vs INAH+INCS | 0.71 (-4.40;5.82) [0.785] |
| OAH vs INCS+OAH | 2.59 (-2.05;7.23) [0.274] |
| INCS vs INCS+OAH | 5.21 (0.92;9.50) [0.018] |
| INAH+INCS vs INCS+OAH | 1.96 (-5.41;9.33) [0.603] |
| **B. Adjusted models ^a^** |  |
| OAH vs INCS | -3.28 (-6.38;-0.18) [0.038] |
| OAH vs INAH+INCS | 1.47 (-3.03;5.97) [0.521] |
| INCS vs INAH+INCS | 2.43 (-3.24;8.10) [0.401] |
| OAH vs INCS+OAH | 1.77 (-2.99;6.53) [0.466] |
| INCS vs INCS+OAH | 4.66 (0.15;9.17) [0.043] |
| INAH+INCS vs INCS+OAH | 0.57 (-7.39;8.53) [0.889] |

INAH=Intranasal antihistamines; INCS=Intranasal corticosteroids; OAH=Oral antihistamines; VAS satisfaction=Visual analogue scale evaluating patients’ satisfaction with their rhinitis treatment; ^a^ Adjusted for age, sex and baseline severity.

**Supplementary Table 7. Levels of the visual analogue scale (VAS) on treatment satisfaction in relation to oral antihistamines in individual countries.** Countries were selected on the basis of having at least 100 observations of VAS satisfaction, something which was only observed for oral antihistamines.

| **Country** | **Regular treatment – median (IQR) [*N* observations]** | **As-needed treatment – median (IQR) [N observations]** |
| --- | --- | --- |
| France | 51 (34-83) [111] | 95 (92-96) [35] |
| Germany | 79 (59-89) [330] | 79 (59-88) [78] |
| Italy | 85 (61-92) [297] | 87 (64-94) [70] |
| Poland | 81 (73-95) [424] | 87 (75-93) [54] |
| Portugal | 80 (59-93) [176] | 79 (65-89) [45] |

**Supplementary Figure 1. Flow diagram illustrating participant selection**
